# Therapy options for adrenal insufficiency and recommendations for the management of adrenal crisis

**DOI:** 10.1007/s12020-021-02649-6

**Published:** 2021-03-04

**Authors:** Hanna Nowotny, S. Faisal Ahmed, Sophie Bensing, Johan G. Beun, Manuela Brösamle, Irina Chifu, Hedi Claahsen van der Grinten, Maria Clemente, Henrik Falhammar, Stefanie Hahner, Eystein Husebye, Jette Kristensen, Paola Loli, Svetlana Lajic, Nicole Reisch, On Behalf of Endo ERN (MTG1)

**Affiliations:** 1 Medizinische Klinik IV, Klinikum der Universität München, Munich, Germany; 2 Developmental Endocrinology Research Group, University of Glasgow, Glasgow, Scotland UK; 3 Department of Molecular Medicine and Surgery, Karolinska Institutet and Department of Endocrinology, Karolinska University Hospital, Stockholm, Sweden; 4 European Patient Advocacy Group for Adrenal Diseases, European Reference Network on Rare Endocrine Conditions (Endo ERN), Endo ERN Coordinating Centre, Leiden, The Netherlands; 5 AdrenalNET, Soest, The Netherlands; 6 Division of Endocrinology and Diabetology, Department of Internal Medicine I, University Hospital of Wuerzburg, University of Wuerzburg, Wuerzburg, Germany; 7 Amalia Children’s Hospital, Department of Pediatric Endocrinology, Radboud University Medical Centre, Nijmegen, The Netherlands; 8 Pediatric Endocrinology Unit, Hospital Vall d´Hebron, Autonomous University of Barcelona, CIBERER, Barcelona, Spain; 9 Department of Clinical Science and K.G. Jebsen Center for Autoimmune Disorders, University of Bergen, and Department of Medicine, Haukeland University Hospital, Bergen, Norway; 10 Division of Endocrinology, San Raffaele Vita-Salute University, IRCCS San Raffaele Hospital, Milan, Italy; 11 Department of Women´s and Children´s Health, Division of Pediatrics, Unit for Pediatric Endocrinology and Metabolic Disorders, Karolinska Institutet/Karolinska University Hospital, Stockholm, Sweden

**Keywords:** Adrenal insufficiency, Congenital adrenal hyperplasia, Adrenal crisis, Glucocorticoid replacement, Hydrocortisone, Stress instructions

## Abstract

Adrenal insufficiency (AI) is a life-threatening condition requiring life-long glucocorticoid (GC) substitution therapy, as well as stress adaptation to prevent adrenal crises. The number of individuals with primary and secondary adrenal insufficiency in Europe is estimated to be 20–50/100.000. A growing number of AI cases are due to side effects of GC treatment used in different treatment strategies for cancer and to immunotherapy in cancer treatment. The benefit of hormone replacement therapy is evident but long-term adverse effects may arise due to the non-physiological GC doses and treatment regimens used. Given multiple GC replacement formulations available comprising short-acting, intermediate, long-acting and novel modified-release hydrocortisone as well as subcutaneous formulations, this review offers a concise summary on the latest therapeutic improvements for treatment of AI and prevention of adrenal crises. As availability of various glucocorticoid formulations and access to expert centers across Europe varies widely, European Reference Networks on rare endocrine conditions aim at harmonizing treatment and ensure access to specialized patient care for individual case-by-case treatment decisions. To improve the availability across Europe to cost effective oral and parenteral formulations of hydrocortisone will save lives.

## Introduction

Thomas Addison first described adrenal insufficiency in 1855. However, it was not until 1948 that a larger quantity of cortisone (so called compound E) was synthesized as a result of the discovery and works by Kendall, Sarett and Reichstein, and it could thereafter be used for treatment of the disease and prevention of adrenal crisis. Individuals with adrenal insufficiency (AI) have reduced or no production of glucocorticoids and in primary adrenal insufficiency this is often associated with a reduction in mineralocorticoid synthesis [1]. In Western countries primary adrenal insufficiency (PAI; adrenal level) is mainly caused by an autoimmune adrenalitis (Mb Addison) and can go in hand with other autoimmune endocrine diseases (polyendocrine syndrome). PAI can also be due to infections, bilateral infarction, metastases, hemorrhages and adrenalectomy, as well as genetic syndromes (e.g., congenital adrenal hyperplasia (CAH), adrenoleukodystrophy) [2]. The main causes of secondary adrenal insufficiency (SAI; pituitary level) are tumors of the pituitary gland, adrenocorticotropic hormone (ACTH) deficiency, and hypophysitis due to increasing use of immune checkpoint inhibitors. Other causes include trauma due to operation, infarction such as Sheehan’s syndrome or infections. Tertiary adrenal insufficiency (hypothalamic level) is probably the most common cause of AI and mostly due to long-term high-dose synthetic glucocorticoid therapy but can also be caused by repressing lesions and certain drugs (opioids) resulting in a prolonged hypothalamic–pituitary–adrenal axis suppression [3, 4].

Physiological cortisol secretion has a distinct diurnal rhythm with a rise over night, a peak upon awakening and decreasing levels during the day and depends on the same diurnal ACTH secretion. This robust pattern is controlled by the interaction of the suprachiasmatic nucleus (SCN) in the hypothalamus as the main circadian pacemaker with the hypothalamus-pituitary-adrenal (HPA) axis, as well as peripheral clocks located in many organs including the adrenals via differential expression of so-called “canonical clock genes” [5]. Glucocorticoids themselves are serving as transducers between these central and peripheral systems [6] and also play a key role in the regulation of multiple body functions, such as metabolism, the stress- and immune response and cognition [7]. Moreover, cortisol is secreted in a pulsatile way with peaks approximately every 3 h, which trigger pulsatile binding to the glucocorticoid receptor (GR) and GR-mediated gene pulsing [8]. This rationale indicates that disruptions in any part of this tightly controlled system can possibly lead to detrimental effects and pathological changes in several body functions [7].

## Current glucocorticoid replacement therapy

Replacement therapy for patients with AI consists of the application of glucocorticoids, and in PAI mostly additional mineralocorticoids. Conventional therapeutic regimens include short-acting substances as hydrocortisone the synthetic form of cortisol and cortisone acetate or synthetic intermediate or long-acting glucocorticoids, such as prednisone, prednisolone, and dexamethasone. Short-acting glucocorticoids should always be used as the primary treatment option. A total of 15–25 mg hydrocortisone (cortisone acetate 20–35 mg) in adults in two to three daily doses and 8 mg/m^2^ body surface/day in children divided in three to four doses, or appropriate equivalent dose if using a synthetic glucocorticoid, is considered an adequate hormone replacement dose, even though the daily production of cortisol is even lower (5–6 mg/m^2^) [9, 10]. In CAH mostly higher glucocorticoid (GC) replacement doses than in primary adrenal insufficiency due to Addison’s disease are necessary to adequately suppress adrenal androgen production. In children with CAH, hydrocortisone in a range of 10–15 mg/m^2^ body surface/day is mostly necessary and in adults 15–25 mg/day [11]. Practical experience shows that glucocorticoid dosing in CAH often is higher than recommended [12, 13]. Mineralocorticoids in PAI are replaced by fludrocortisone at a dose of 0.05–0.2 mg/day [9, 11]. It is important to know that hydrocortisone exerts potent mineralocorticoid action while prednisolone only has reduced mineralocorticoid activity and dexamethasone none at all.

Hormone replacement shows substantial heterogeneity across Europe, but hydrocortisone twice or thrice daily is most widely used [14]. Chronic cortisol replacement with long-acting synthetic glucocorticoids is less desirable as it exerts unfavorable night time glucocorticoid activity as a consequence of the longer biological half-lives and has limited options for dose titration. Longer-acting GC replacement regimen have shown to be associated with more adverse effects [13, 15–18].

However, even today in many countries, including European countries, hydrocortisone is not available, but cortisone acetate (e.g., Italy and Norway) or longer-acting synthetic GCs need to be used. In some situations long-acting synthetic glucocorticoids as prednisolone may be preferred over hydrocortisone, e.g., for compliance reasons, better control or suppression of adrenal androgens in CAH.

Undoubtedly, excess glucocorticoid replacement affects quality of life and causes multiple morbidities and increased mortality [19–22], which can only partially be explained by a higher incidence of adrenal crises in this group of patients [23]. Research in recent years revealed compelling evidence of side effects of dysregulated glucocorticoid rhythmicity highlighting that the mode of glucocorticoid replacement matters [24]. Patients with long-term supraphysiological GC replacement have been found to be at risk for a higher incidence of infections, especially those of the upper airways and gastrointestinal tract, and show an increased rate of hospitalization and use of antimicrobial substances [25]. Bancos et al. [26] observed that patients with AI under standard hydrocortisone therapy present with significantly impaired NK-cell cytotoxicity along with a reduced expression of NK-cell-specific surface receptors. In addition, ratios of classical to non-classical monocytes were shown to be inversed, with higher levels of classical CD14 + CD16- monocytes under traditional hydrocortisone treatment. Further studies are required to determine whether patients with AI have an increased risk of infections. Non-physiological and supra-physiologic glucocorticoid substitution therapy may also be associated with an increase in cardiovascular and metabolic morbidity [27–30]. A Swedish population-based cohort study in patients with CAH showed higher incidence of cardiovascular and metabolic disorders compared to healthy controls [28]. Especially in females with PAI the risk of ischemic heart disease and cardiovascular disease was increased and associated with higher glucocorticoid replacement doses [30]. Besides these substantial morbidities, the dysregulation of the cortisol circadian profile is negatively impacting on the capability, endurance, and sleep quality resulting in impaired physical and mental health, as well as reduced quality of life [31, 32].

## New developments in glucocorticoid replacement therapies

In attempting to prevent or minimize these detrimental effects caused by circadian changes on health in patients with AI, further treatment options aiming at mimicking normal circadian cortisol rhythm have been developed.

Plenadren® (Takeda) was approved for treatment of AI in adults in 2011. The main pharmacological principle is a dual-release system resulting from an outer layer providing immediate release and an inner retard formulation [33]. Comparison of once-daily Plenadren® and conventional twice-daily hydrocortisone therapy indicated a smoother cortisol profile up to 4 h after Plenadren® intake mimicking the physiological diurnal cortisol levels with the exception of a rise in cortisol levels prior to awakening [34], as well as an overall reduction of daily cortisol exposure [35]. A switch to dual-release preparations (Plenadren®) reduced the levels of monocytes to the levels found in untreated controls and lead to a significant reduction in body weight, body mass index (BMI), and HbA1c [36, 37]. It still remains unclear whether these findings represent a better physiological circadian profile or are the result of a lower daily dose of hydrocortisone equivalent [38, 39]. The economic burden of an ~10 times higher price of Plenadren® versus conventional hydrocortisone also needs to be taken into account and limits the availability. It may, however, be used in certain subgroups of patients, e.g., in patients with the greatest risk of metabolic comorbidities and in patients with poor therapeutic compliance.

Chronocort® (Diurnal, UK) is a modified-release preparation, currently undergoing the approval process in Europe for treatment of CAH in adults. The drug consists of multiple micro-crystals covered by a polymer sheathing, enabling a delayed and sustained release. It is used according to a “toothbrush-regimen” (1/3 of the daily dose at 7 a.m. and 2/3 of the daily dose at 11 p.m.) and results in nearly physiological cortisol levels throughout the entire day including the overnight rise and the morning peak in cortisol [40]. This is especially important for patients with CAH as it prevents the ACTH-driven excess production of adrenal androgens and thus reduces the disease impact on growth, puberty, and fertility [41]. Data from the phase III trial including 122 patients with classic CAH due to 21-hydroxylase deficiency (21OHD) showed that both, modified-release hydrocortisone and conventional glucocorticoid treatment regimen, achieved improved hormonal control at 6 months. The study found better biochemical control on Chronocort® versus conventional glucocorticoid therapy, with lower 17-hydroxyprogesterone standard deviation score (SDS) at 4 and 12 weeks, but only between 07:00 h and 15:00 h at 6 months, thus failing its primary outcome at 6 months. Further observations included a lower number of adrenal crises per year, women restarting menstruation and two partner pregnancies with Chronocort® therapy [42].

Although Chronocort® appears to offer a good imitation of the circadian pattern of cortisol secretion, it still misses the pulsatile secretory pattern. Subcutaneous infusion of glucocorticoids via specialized pumps have been shown to enable reproduction of near physiological patterns of both circadian and ultradian rhythmicity imitating the production of individual pulses with an interval of 80–110 min and plasma peaks and troughs every 3 h as previously described [8, 43–45]. Economic reasons and the complexity of having a pump as well as absent evidence for the superiority of such treatment reserve this treatment for special cases, which have so far prevented its use in clinical practice.

A particular challenge of GC replacement is accurate dosing in low-dose ranges for children. Until recently this had to be done by crushing hydrocortisone tablets or pharmacy compounded capsules as licensed preparations of hydrocortisone have only been available as 10 mg tablets in Europe and 5 mg tablets in the United States. However, up to 25% of pharmacy compounded capsules have been shown not to fulfill the acceptance criteria of the European Pharmacopeia of uniformity of net mass or drug content or showed inadequate labeling [46]. To overcome this, in Europe hydrocortisone granules for children and adolescents (Alkindi®) now have been licensed in doses of 0.5, 1, 2, and 5 mg [47]. The draw-back of these granules is the high cost compared to conventional hydrocortisone tablets or compounded capsules, which may limit their availability in different countries. In infants it may also be difficult to administer the granules since they cannot be dissolved.

In May 2020 in the Netherlands, the Dutch Medicines Evaluation Board (CBG) has approved a registration for a new series of hydrocortisone tablets. They are covered with a small film to neutralize the bad taste and all have their own color. Currently 1 mg (white), 5 mg, and 10 mg (red) are available. At the moment (November 2020), the other two tablets (2 mg and 3 mg) are being under evaluation by the Dutch Medicines Evaluation Board (CBG).

## Adrenal crisis: improving prevention of one of the most significant morbidity factors in AI

Besides daily routine hormone replacement requiring improvement and the need for further research regarding long-term health benefits potentially offered by these novel therapeutic options, adrenal crisis (AC) prevention, one of the most significant morbidity factors in AI, is still a challenge. With an incidence of 5–10 adrenal crises/100 patient years and a mortality rate of 0.5/100 years [48], prevention, quick diagnosis, and correct treatment is essential [49]. It should be noted that both symptoms and signs of adrenal crisis may be more difficult to interpret in the old patient and in young children [50].

In particular, the current SARS-CoV-2 pandemic and the risk of COVID-19 infection emphasizes the importance of education on this topic. Currently, there is no evidence that patients with AI are at an increased risk of acquiring COVID-19 or are prone to a severe course of COVID-19 infections. Adrenal crises are rare events in children and when examined in children with rare conditions such as CAH, they are even rarer [51]. However, it cannot be stressed enough that there is a need to collect real-world data on adrenal crises and sick day episodes in such a way that it can be linked to changes in therapies or other events such as the COVID-19 pandemic. Registry platforms such as the I-CAH Registry (https://home.i-cah.org) have the potential to serve this purpose over the longer term. Cases of patients with AI and COVID-19 infection are currently being ascertained by a joint initiative of ESE and EndoERN through a surveillance project that is being performed on EuRRECa’s e-REC platform (https://eurreca.net) and is open to all centers worldwide (https://www.ese-hormones.org/research/rare-disease-covid-19-task-force/). The current situation also emphasizes the need for standardized patient education to prevent adrenal crises in any infection [52–55]. AI is a rare disease and health care professionals may not always be familiar with the optimal management of adrenal crisis in these patients [56]. Therefore, a well-trained patient (and/or relative) is the key success factor for prevention and management of an adrenal crisis.

Steroid emergency cards for children and adults already have been successfully harmonized across Europe (Fig. 3) and are available in the respective national language on one side and in English on the other (https://adrenals.eu/emergency-card/) [57].

Patients with AI should regularly be educated on the use of sick day rules, emergency equipment and empowerment [56]. Sick day rules, e.g., include doubling or tripling of standard glucocorticoid daily doses, application of hydrocortisone every 6 h with increasing amount upon clinical deterioration and emergency injection of hydrocortisone (e.g., Solu-Cortef®, Hydrocortisone 100-Rotexmedica®, Hydrocortisone Panpharma 100®, Actocortina 100 mg®) either intramuscularly or intravenously [49] (https://adrenals.eu/stress-instructions/). Although off-label, subcutaneous hydrocortisone application can be used in adults instead of i.m. injection, the pharmacokinetics during real life emergency use should be evaluated further [58]. In some European countries, a hydrocortisone solution for i.v. or i.m. injection (e.g., Solu-Cortef®/Hydrocortisone 100-Rotexmedica®/Hydrocortisone Panpharma 100®/Actocortina 100 mg®) is not available, and other glucocorticoids have to be used instead (https://endo-ern.eu/specific-expertise/adrenal/existing-guidelines-consensus-statements/). There is thus a need for distributing and providing both oral and parenteral formulations of hydrocortisone at a reasonable cost across Europe. This will save lives and reduce long-term morbidity.

**Fig. 1:**
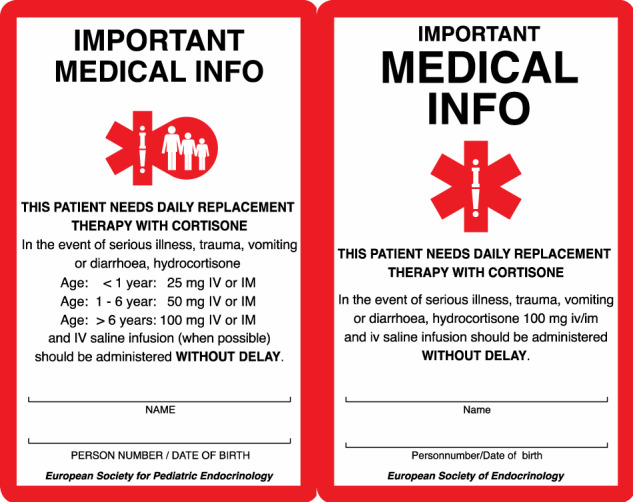
European steroid emergency card for children (left) and adults (right) in the English version (https://adrenals.eu/emergency-card/)

**Fig. 2:**
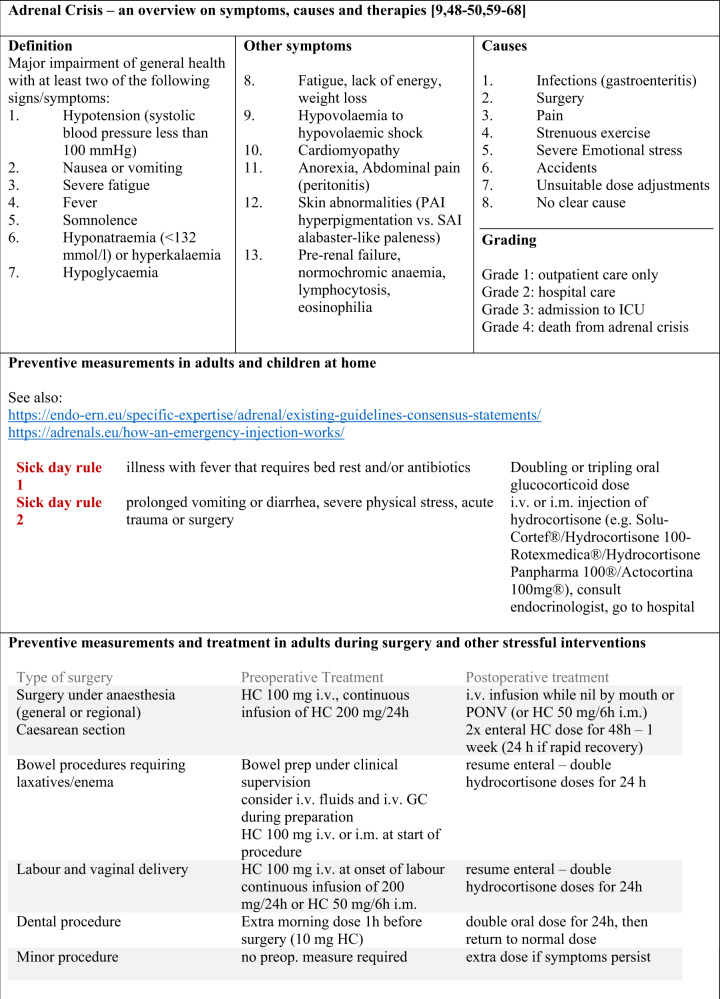
Overview on symptoms, causes and preventive therapies of adrenal crisis. ICU intensive care unit, PAI primary adrenal insufficiency, SAI secondary adrenal insufficiency, GC glucocorticoid, i.m. intramuscular, i.v. intravenously, s.c. subcutaneously, PONV postoperative nausea and vomiting, HC hydrocortisone, mg milligram, h hour [9, 48–50, 59–68]

**Fig. 3:**
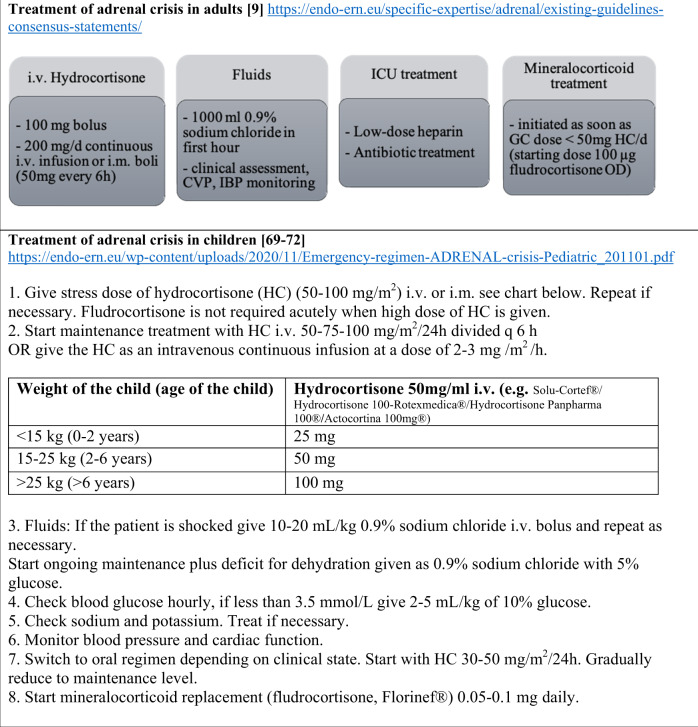
Summary of treatment of adrenal crisis in adults and children in the hospital setting. i.v. intravenously, i.m. intramuscular, h hour, CVP central venous pressure, IBP invasive blood pressure, ICU intensive care treatment [9, 69–72]

It is essential that patients are equipped at any time with an emergency card (Fig. 1) and an emergency kit for self-injection. They should always have a supply of medication of at least three months including a sick day package of hydrocortisone tablets. Further information on prevention and treatment of adrenal crises in children and adults is summarized in Figs. 2 and 3 or can be found by using the following link (https://endo-ern.eu/specific-expertise/adrenal/existing-guidelines-consensus-statements/).

## Conclusion

Conventional immediate-release hydrocortisone remains the gold standard glucocorticoid replacement therapy in patients with adrenal insufficiency. However, dual- and modified-release hydrocortisone and other, still experimental approaches can already now be an alternative for those with poor quality of life and/or metabolic comorbidities. Larger, long-term outcome-based trials are required to assess the benefits of newer preparations against current treatment practice. There is still a need with regards to harmonization of treatment and patient education, as well as access to medications and specialized patient care across Europe. This is a dedicated aim of the European Reference Network on Rare Endocrine Conditions (EndoERN).
